# Autonomic dysfunction and exercise intolerance in post-COVID-19 - An as yet underestimated organ system?

**DOI:** 10.1016/j.ijchp.2023.100429

**Published:** 2023-12-14

**Authors:** F. Schwendinger, V.N. Looser, M. Gerber, A. Schmidt-Trucksäss

**Affiliations:** a Division of Sports and Exercise Medicine, Department of Sport, Exercise and Health, University of Basel, Grosse Allee 6, 4052 Basel, Switzerland; b Division of Sports Science, Department of Sport, Exercise and Health, University of Basel, Grosse Allee 6, 4052 Basel, Switzerland; c Department of Clinical Research, University Hospital Basel, University of Basel, Schanzenstrasse 55, 4056 Basel, Switzerland

**Keywords:** Long COVID, Exercise capacity, Central nervous system, Exercise neuroscience, Mental health

## Abstract

Individuals recovering from COVID-19 often present with persistent symptoms, particularly exercise intolerance and low cardiorespiratory fitness. Put simply, the Wasserman gear system describes the interdependence of heart, lungs, and musculature as determinants of cardiorespiratory fitness. Based on this system, recent findings indicate a contribution of peripheral, cardiovascular, and lung diffusion limitations to persistent symptoms of exercise intolerance and low cardiorespiratory fitness. The autonomic nervous system as an organ system involved in the pathophysiology of exercise intolerance and low cardiorespiratory fitness, has received only little attention as of yet. Hence, our article discusses contribution of the autonomic nervous system through four potential pathways, namely alterations in (1) cerebral hemodynamics, (2) afferent and efferent signaling, (3) central hypersensitivity, and (4) appraisal and engagement in physical activity. These pathways are summarized in a psycho-pathophysiological model. Consequently, this article encourages a shift in perspective by examining the state of the pulmonary and cardiovascular system, the periphery, and auxiliary, the autonomic nervous system as potential underlying mechanisms for exercise intolerance and low cardiorespiratory fitness in patients with post-COVID-19.

## Introduction

Up to one-third of all adults recovering from COVID-19, including asymptomatic cases, report persisting symptoms (Gebhard et al., 2021). Exercise intolerance and consequently low cardiorespiratory fitness (CRF) are some of the common symptoms (Gebhard et al., 2021; Schwendinger, Knaier, Radtke et al., 2023). Only recently, researchers reviewed pertinent studies investigating exercise intolerance and possible underlying mechanisms in patients post-COVID-19 based on the three Wasserman gears – pulmonary system, cardiovascular system, and periphery (Schwendinger, Knaier, Radtke et al., 2023). The gear system illustrates the interdependency of the three organ systems and their modulator role for CRF (Wasserman, 1988). It was concluded that deconditioning may not be the sole cause of low CRF (Schwendinger, Knaier, Radtke et al., 2023). Cardiovascular and lung diffusion limitations secondary to peripheral parameters such as vascular, mitochondrial, or muscular impairment seem to contribute to the condition in most patients (Schwendinger, Knaier, Radtke et al., 2023). As seen in individuals with persistent COVID-19 symptoms, with lower CRF compared to those without symptoms, potentially due to dysfunctional breathing, chronotropic incompetence, and peripheral involvement (Durstenfeld et al., 2022).

The autonomic nervous system (ANS) has been shown to be another organ system that may limit exercise capacity in some populations (Gagnon et al., 2012; Noakes, 2011; Swart et al., 2012). Yet, to the best of our knowledge, this organ system has been left out in the search of possible underlying mechanisms for low CRF in patients with post-COVID-19. While fatigue and cognitive impairment are amongst the most frequent and constraining symptoms of post-COVID-19, their role in exercise intolerance and low CRF has not yet been decrypted (Ceban et al., 2022). Thus, this article aims to (1) provide a balanced, evidence-based discussion on how the ANS may contribute to low CRF in patients post-COVID-19 and (2) introduce a psycho-pathophysiological model summarizing potential underlying factors. Importantly, this work does NOT propose the ANS as the sole underlying mechanism of low CRF post-COVID-19 but rather as one potential contributor in a complex pathophysiological system (Schwendinger, Knaier & Schmidt-Trucksäss, 2023).

## The autonomic nervous system as a valuable addition to the Wasserman gear system

### Models of exercise (in)tolerance

The potential role of the ANS as a limiting organ system of exercise performance is increasingly being recognized (Gagnon et al., 2012; Noakes, 2011; Swart et al., 2012). Various models attempted to explain the complex underlying mechanisms of fatigue. The “Central Governor” model (Noakes, 2011; Noakes et al., 2001) has been heavily studied by researchers around Timothy D. Noakes and is highly disputed among the scientific community (Inzlicht & Marcora, 2016; Marcora, 2008; Weir et al., 2006). The model describes a subconscious central neural governor which may affect neural activation of locomotor muscles during maximal exercise exertion as a protective mechanism (Marcora, 2008; Noakes et al., 2001). According to the theory, the subconscious central neural governor is able to bypass voluntary control (discussed in more detail elsewhere (Marcora, 2008)). However, growing evidence accumulated over the last decade refutes this model and proposes alternative explanations (Inzlicht & Marcora, 2016; Weir et al., 2006). Marcora (2008), for instance, proposed a psychobiological model in which neural activation is governed solely by voluntary control. Voluntary control, in turn, may be influenced by motivation and/or sensory information via perceived exertion (Marcora, 2008). This model offers an emphasis on both peripheral and central factors which might be involved in the development of fatigue (Marcora, 2008). While there is ongoing debate regarding the extent to which fatigue is under voluntary control, evidence suggests that the underlying mechanisms of fatigue may be task-dependent (Weir et al., 2006). As a consequence, investigating the causes of task failure in view of the specific task-dependent factors (i.e. muscle fiber composition, type of contraction, environment) may be a desirable strategy (Weir et al., 2006). Moreover, when applying the concept of task dependency to patient populations, in this case patients with post-COVID-19, information on the current health condition are vital and need to be factored in.

**Fig. 1:**
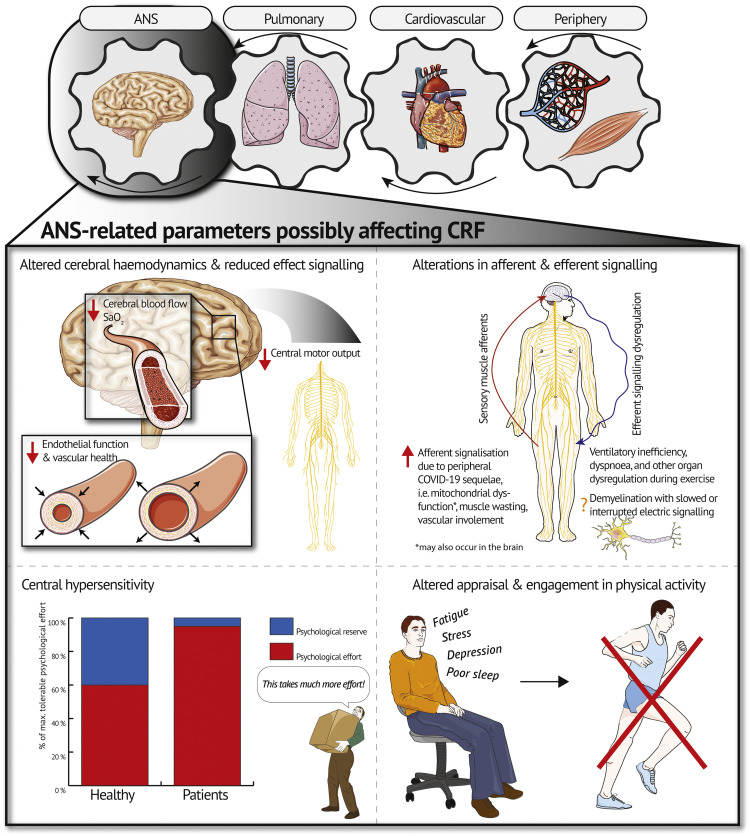
Overview of pathways through which the autonomic nervous system (ANS) might contribute to low cardiorespiratory fitness (CRF) and exercise intolerance in patients with post-COVID-19. Abbreviations: SaO2, arterial oxygen saturation in the brain.

Based on the current state of research investigating exercise intolerance post-COVID-19 (Schwendinger, Knaier, Radtke et al., 2023), we believe it is time to discuss the ANS as a contributor to exercise intolerance in patients with post-COVID-19. This adds the ANS as a fourth gear to the traditional Wasserman gear system (Wasserman, 1988) as a potential modulator of CRF (see Fig. 1). Below, we will discuss four potential pathways through which the ANS may contribute to exercise intolerance and low CRF in patients post-COVID-19.

### Altered cerebral hemodynamics

Recent evidence showed altered cerebral hemodynamics at rest in patients with post-COVID-19 (Ajčević et al., 2023; Qin et al., 2021). Indirect brain injury resulting from a post-viral immune response-related inflammatory storm may be the underlying mechanism leading to the observed alterations in cerebral hemodynamics (Ajčević et al., 2023; Qin et al., 2021). This corresponds to findings from patients with myalgic encephalomyelitis/chronic fatigue syndrome (ME/CFS), showing altered cerebral hemodynamics in form of lower cerebral blood flow compared to healthy controls (Boissoneault et al., 2019; van Campen et al., 2021). Additionally, cerebral blood flow at rest was inversely linked to fatigue levels and ME/CFS severity in these patients (Boissoneault et al., 2019; van Campen et al., 2021). This underscores the potential contribution of blood flow to the overall fatigue experienced by patients with ME/CFS which might also apply to patients with post-COVID-19.

During exercise, the reduced perfusion of the brain may persist or even worsen which in turn, might cause excessive fatigue (Boissoneault et al., 2019) and reduced motor unit recruitment (Secher et al., 2008; Verges et al., 2012), resulting in premature exercise cessation (Noakes, 2011). Cerebral endothelial dysfunction could be an underlying contributing factor of such impaired cerebral blood flow (Kim et al., 2015). Evidence in patients with type 2 diabetes indicates lower cerebral blood flow and oxygenation at higher relative exercise intensities compared to healthy counterparts due to impaired endothelial function and inability to increase cardiac output, resulting in reduced exercise capacity (evident by lower maximum power) and possibly higher perceived exertion (Kim et al., 2015). As proposed by the authors, CRF may be limited due to lower cerebral capillary oxygenation and brain mitochondrial O_2_ tension in patients with type 2 diabetes compared to healthy controls (Kim et al., 2015). A drop in brain mitochondrial O_2_ tension is associated with increased cerebral lactate production and reduced CRF (Rasmussen et al., 2007). The resulting mismatch between oxygen delivery and neuronal activity as well as the altered metabolic milieu may result in impaired activation of exercising muscles (Kim et al., 2015; Rasmussen et al., 2007). This could support the role of impaired cerebral oxygenation in the development of central fatigue and consequently low CRF. However, contrary findings also exist. Subudhi et al. (2011) demonstrated that although end-tidal pCO_2_ was raised in a CO_2_-clamp trial (as a vasodilator) during incremental exercise in road cyclists leading to higher cerebral blood flow, exercise performance did not improve (Subudhi et al., 2011). While a supernormal cerebral blood flow at peak exercise may not improve exercise performance in healthy individuals (Subudhi et al., 2011), further reductions in blood flow and oxygen saturation, as possibly encountered in patients with impaired endothelial function (e.g. some patients post-COVID-19), might limit central motor drive (Kim et al., 2015; Rasmussen et al., 2010). However, future studies with patients post-COVID-19 has to verify this hypothesis by measuring cerebral oxygen saturation during cardiopulmonary exercise testing (CPET) e.g. using near-infrared spectroscopy or comparing between-group differences in cerebral blood flow at set times following exercise using arterial spin labelling MRI (Boissoneault et al., 2019). The latter would provide insights on potential occurrence of delayed recovery of cerebral blood flow compared to healthy controls as found after tilt testing in patients with ME/CFS (van Campen et al., 2021). Lastly, coagulopathy, which is well-documented in patients with post-COVID-19, should be considered as a contributing factor (Davis et al., 2023). Micro clots forming in the blood may induce damaging of the vessels (Davis et al., 2023) and consequent impairments in oxygen supply to the brain and working musculature.

### Alterations in afferent and efferent signaling

There is growing evidence for an autoimmune-mediated ANS dysfunction that may play a role in the pathogenesis of some patients with post-COVID-19 similar to ME/CFS or postural orthostatic tachycardia syndrome (Dotan et al., 2022). There is a multitude of symptoms/conditions caused by ANS dysfunction which may lead to low CRF and exercise intolerance such as fatigue exacerbation, orthostatic intolerance, or dyspnea (Dotan et al., 2022). However, the exact mechanisms may differ between phenotypes of post-COVID-19 (Gentilotti et al., 2023).

An interesting study in chronic obstructive pulmonary disease (COPD) suggested that ANS dysfunction through enhanced levels of sensory muscle afferents might be another potential pathway inducing low CRF and exercise intolerance (Gagnon et al., 2012). When blocking sensory muscle afferents in patients, Gagnon et al. (2012) recorded greater time to exhaustion and thus a higher level of muscular fatigue than without blocking. This was accompanied by a reduced breathing frequency and ventilation with, in turn, improved ventilatory efficiency (Gagnon et al., 2012). These findings are relevant due to the symptom overlap with some patients suffering from post-COVID-19, i.e. dyspnea and ventilatory inefficiency (Schwendinger, Knaier, Radtke et al., 2023). A previous review demonstrated that the periphery might contribute to low CRF due to muscle wasting, vascular involvement, as well as mitochondrial dysfunction (Schwendinger, Knaier, Radtke et al., 2023). We, therefore, propose that these peripheral alterations could lead to exercise intolerance by enhanced afferent signalization during exercise potentially causing ventilatory inefficiency, dyspnea, and even dysregulation of other organ systems as seen in COPD (see Fig. 1) (Gagnon et al., 2012). As discussed by Gagnon et al. (2012), this mechanism could potentially be more relevant in patient populations with an earlier reliance on anaerobic metabolism than in healthy individuals as it is the case in patients with post-COVID-19 (Schwendinger, Knaier, Radtke et al., 2023). Díaz-Resendiz et al. (2022) provided evidence of mitochondrial dysfunction in these patients, preventing adequate utilization of aerobic metabolism. Larger dependence on anaerobic pathways for energy supply consequently leads to an increased accumulation of hydrogen ions. Metabolic acidosis, in turn, might enhance afferent signaling since synaptic function is highly dependent on intra- and extracellular pH gradients (Sinning & Hübner, 2013). This might also occur in the brain. Studies using imaging electromyography during exercise in patients post-COVID-19 and matched counterparts without a history of COVID-19 might shed light on the plausibility of this mechanism (Gagnon et al., 2012).

The concepts of altered cerebral hemodynamics and enhanced afferent signaling might be contrasting. Impaired oxygen supply to the brain would induce a subsequent reduction in motor unit recruitment, whereas enhanced afferent signaling would lead to ANS dysregulation (potentially also increased efferent signaling to provoke respiration (Gagnon et al., 2012)). However, based on the available literature, both pathways may be applicable depending on the individual and the phenotype of post-COVID-19 (e.g., chronic fatigue-like syndrome, respiratory syndrome, chronic pain syndrome, and neurosensorial syndrome) (Gentilotti et al., 2023).

Structural changes of the ANS by demyelination of central glia cells with slowed or interrupted electric signaling have also been discussed in the literature as a process leading to neurological complaints in patients with post-COVID-19 (Davis et al., 2023). Whether this process could also contribute to exercise intolerance and low CRF is currently unknown. Reduced efferent neural drive and afferent feedback as consequence of this may be worthwhile investigating in the future.

### Central hypersensitivity

The ANS is proposed to use two sensations to regulate exercise performance, namely feedback on physical strain and psychological effort required to sustain a task (Swart et al., 2012). Considering the high psychological burden imposed on patients with post-COVID-19 (Han et al., 2022; Schaeffer et al., 2022; Shanbehzadeh et al., 2021), it seems plausible that changes in central regulations might lead to an exacerbation of psychological effort during high-intensity exercise – herein referred to as central hypersensitivity. Central hypersensitivity might be mediated through fatigue (Schaeffer et al., 2022; Swart et al., 2012). Physiological factors such as systemic inflammation, immune activation, and mitochondrial damage have been shown to worsen fatigue and as a result may amplify the experienced psychological effort (Morris et al., 2015). This has been demonstrated in patients with post-COVID-19 (Ajčević et al., 2023; Qin et al., 2021) and patients with ME/CFS (Boissoneault et al., 2019; van Campen et al., 2021). The ceiling of maximum tolerable physical and psychological effort might thus be reached earlier, i.e. at a lower exercise intensity compared to healthy individuals with similar CRF (see Fig. 1). For some individuals, this might even be the case during activities of daily life. Quantifying physical and psychological effort separately during CPET (i.e. Borg scale for physical and Task Effort and Awareness scale for psychological effort) (Swart et al., 2012) in both patients with post-COVID-19 and healthy matched controls might, therefore, be beneficial.

## Altered engagement and appraisal of physical activity

Physical activity (PA) is the central modifiable parameter to maintain and improve CRF (Lavie et al., 2019). Besides, being regularly active comes along with numerous beneficial effects on physical and mental health (Lavie et al., 2019). Along with exercise intolerance and low CRF, fatigue and cognitive impairment are some of the most frequently reported post-COVID-19 symptoms (Ceban et al., 2022). Rudroff et al. (2020) define post-COVID-19 fatigue as “the decrease in physical and/or mental performance that results from changes in central, psychological, and peripheral factors due to the COVID-19 disease”. A meta-analysis examining the impact of fatigue and cognitive impairment in patients with post-COVID-19 reported that up to 28.2 % of patients remained unable to partake in recreational activities 12 weeks post-infection (Ceban et al., 2022). Therefore, a plausible pathway through which chronic fatigue may impact CRF post-COVID-19 is via altered capacity to engage in PA (see Fig. 1). Furthermore, low CRF may be accompanied by a fatigue-mediated modification of evaluation of exertional symptoms as seen in higher dyspnea intensity ratings as well as heightened anxiety, distress, depression and functional disability attributed to dyspnea (Schaeffer et al., 2022). These psycho-physiological modifications indicate a higher psychological burden in individuals suffering post-COVID-19 fatigue (Schaeffer et al., 2022).

Psychiatric disorders such as depression, anxiety disorders, post-traumatic stress disorder, sleep disturbances, and resulting low health-related quality of life are prevalent among patients post-COVID-19 and even more frequent in those with severe COVID-19 (Han et al., 2022; Shanbehzadeh et al., 2021). These disorders often present along with low CRF (Kandola et al., 2019). Several studies in patients with depression, stress, or poor sleep have furthermore reported impaired PA as a consequence of these conditions, emphasizing bi-directionality (Roshanaei-Moghaddam et al., 2009). Recent findings suggest a strong association between systemic inflammation during acute COVID-19 and post-viral severity of depressive symptoms in patients with post-COVID-19 (Mazza et al., 2021). Higher rates of depressive symptoms showed a strong influence on neurocognitive functioning resulting in decreased general cognitive functioning, impaired memory performance, as well as executive and visuospatial abilities in patients with higher depression scores (Mazza et al., 2021). As a result, the psychological burden imposed by persistent post-COVID-19 symptoms might induce and reinforce the alteration of engagement, as well as appraisal of PA in patients, preceding to exacerbation of exercise intolerance long-term.

Finally, pre-existing mental and physical conditions might be considered a contributor to post-COVID-19. Schaeffer et al. (2022) stress the need to assess post-COVID-19 in view of conditional dependency, which includes determinants of fatigue associated with the task performed, the environment it is performed in, and an individual´s mental and physical capacity. Female sex, pre-existing psychopathology or psychiatric history have been reported to be strong risk factors for the development of neurocognitive impairment and depression in post-COVID-19, suggesting a potential exacerbation of pre-existing neuropsychological vulnerability (Schaeffer et al., 2022). In terms of context, social, economic, and cultural considerations concerning the psychological burden should not be neglected. Social and economic consequences of the pandemic are to be considered as strong psychosocial stressors exerting pro-inflammatory properties which, in turn, further augment the risk of manifestation of post-COVID-19 and associated psychiatric and neurocognitive symptoms (Mazza et al., 2021; Schaeffer et al., 2022). Early identification of individuals suffering from neurocognitive and psychiatric symptoms post-COVID-19 is critical to provide adequate therapeutic measures and promote PA as such.

## Conclusion and perspective

In this article, we encourage a broadening of perspective in the search for possible underlying mechanisms of exercise intolerance and low CRF in patients with post-COVID-19. We highlight that the ANS might contribute to exercise intolerance via one or several of the following pathways: (a) impaired oxygen supply to the brain and a subsequent reduction in motor unit recruitment, (b) peripheral alterations (e.g. muscle wasting, vascular involvement, as well as mitochondrial dysfunction) as well as structural changes of the ANS by demyelination leading to altered afferent and efferent signalization and successive dysregulation of the ANS, (c) an exacerbation of psychological effort during exercise, and (d) a heightened psychological burden associated with fatigue and neurocognitive impairment post-COVID-19, which may impair engagement and appraisal of PA and consequently CRF. The ANS might thus be the missing gear in the Wasserman gear system deserving more attention in future research on post-COVID-19. Importantly, mechanisms through which the ANS may contribute to low CRF and exercise intolerance may differ depending on post-COVID-19 phenotype (Gentilotti et al., 2023). Also of note, the long-term contribution and interaction of the herein discussed mechanism may vary and need further investigation.

This perspective piece focusses on mechanisms contributing to factors of post-COVID-19 contributing to reduced CRF and acute intolerance during exercise, excluding conditions which occur following exercise such as PEM. However, we acknowledge the potential psychophysiological contribution of PEM to exercise-related symptoms in patients post-COVID-19. This article may constitute a point of departure for future studies in post-COVID-19 and should be interpreted alongside available empirical evidence of the underlying mechanisms of pathophysiology in post-COVID-19 (Davis et al., 2023; Durstenfeld et al., 2022; Schwendinger, Knaier, Radtke et al., 2023).

## Author contributions

FS & VNL conceptualised and drafted the manuscript. MG & AST critically revised the manuscript. All authors read and approved the final version.

## Funding

None to declare.

## Declaration of Competing Interest

The authors declare that they have no known competing financial interests or personal relationships that could have appeared to influence the work reported in this paper.
